# Supplementary material for: Sanctuary for vulnerable Arctic species at the Borealis Mud Volcano
Source: Nat Commun. 2025 Jan 27;16:504. doi: 10.1038/s41467-024-55712-x (PMC11772567; doi:10.1038/s41467-024-55712-x)

Sanctuary for vulnerable Arctic species at the Borealis Mud Volcano

Giuliana Panieri[1], Claudio Argentino[1], Alessandra Savini[2], Bénédicte Ferré[1], Fereshteh Hemmateenejad[2], Mari Heggernes Eilertsen[3], Rune Mattingsdal[4], Sofia P. Ramalho[5], Tor Eidvin[6], Sarah Youngs[7], Beckett Colson[7], Anna P. M. Michel[7], Jason Kapit[7], Denise Swanborn[8], Alex D. Rogers[9,10], Ines Barrenechea Angeles[1], Stéphane Polteau[11], Dimitri Kalenitchenko[1,12], Stefan Buenz[1], Adriano Mazzini[11,13]

[1] Department of Geosciences, UiT - The Arctic University of Norway, 9037 Tromsø, Norway
[2] Department of Earth and Environmental Sciences (DISAT), University of Milano Bicocca, Milan, 20126, Italy
[3] Department of Biological Sciences and Centre for Deep Sea Research, University of Bergen, 5006, Bergen, Norway
[4] Norwegian Offshore Directorate, 9406 Harstad, Norway
[5] Centre for Environmental and Marine Studies (CESAM) & Biology Department, University of Aveiro, 3810-193, Aveiro, Portugal
[6] Dronningåsen 14, Stavanger, NO-4032, Norway
[7] WHOI, Woods Hole Oceanographic Institution, Woods Hole, MA 02543, USA
[8] Minderoo-UWA Deep-Sea Research Centre, University of Western Australia, 6009, Perth, Australia
[9] Ocean Census, Begbroke Science Park, Oxfordshire, OX5 1PF, United Kingdom
[10] National Oceanography Centre, European Way, Southampton, SO14 3ZH, United Kingdom
[11] Reservoir Technology Department, Institute for Energy Technology, 2007, Kjeller, Norway
[12] LIttoral ENvironnement et Sociétés (LIENSs), La Rochelle Université, Bâtiment ILE, La 17000 Rochelle, France
[13] Department of Geosciences, University of Oslo, 0371, Oslo, Norway

Supplementary Figures

Supplementary Figure 1. Seismic random line crossing the *Borealis* mud volcano from 3D-seismic dataset SPE16M01. Directly below the mud volcano, in the centre of the crater, an acoustic chimney is observed with several associated bright amplitude anomalies interpreted as shallow gas.

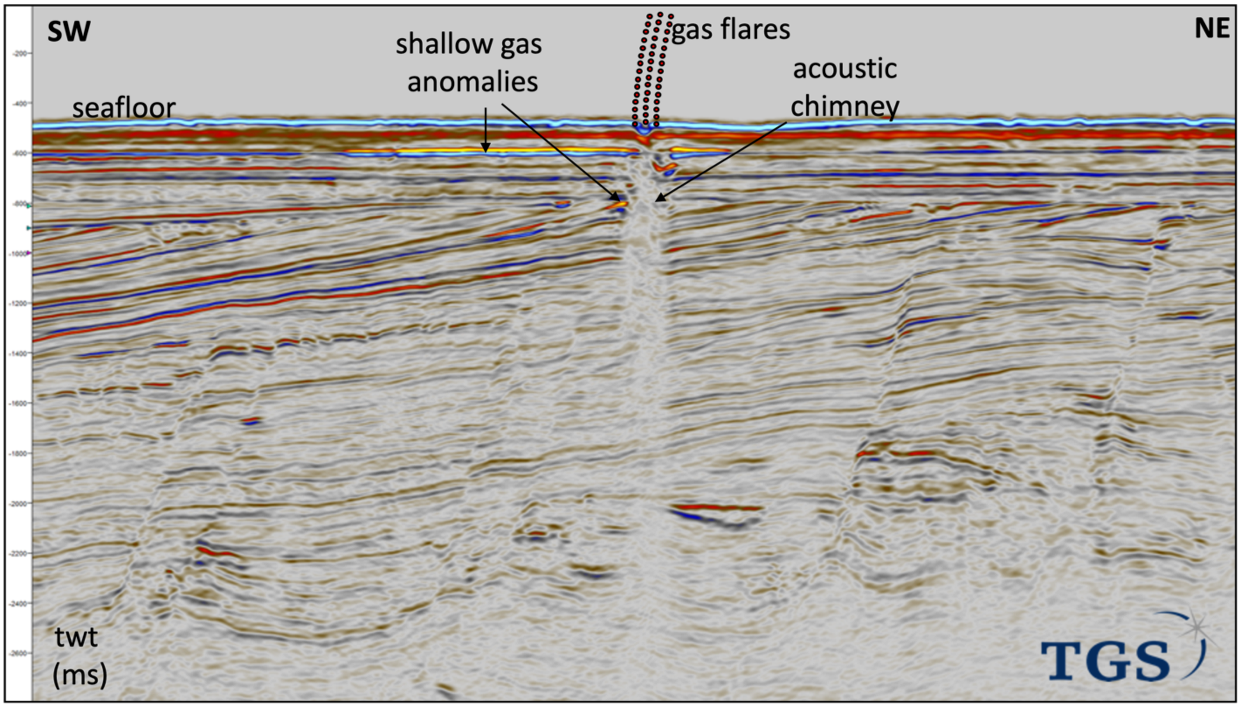

Supplementary Figure 2. WCD (Water Colum Data)-line 0341 across the Borealis mud volcano site (EM710). a Fan view of gas flares in the water column at the site of Dive 13, 14, 15 and 16. Several acoustic gas flares, representing streams of gas bubbles, are imaged from the seafloor and up through the water column, some of them almost reaching the sea surface. The depression at the seafloor at the location of the largest flare observed with ROV dives is indicated with a yellow dotted oval, also in B. **b** R-Stack along ship track crossing the *Borealis* mud volcano site showing biological noise in the water column most likely fish school also observed during the ROV visual survey.

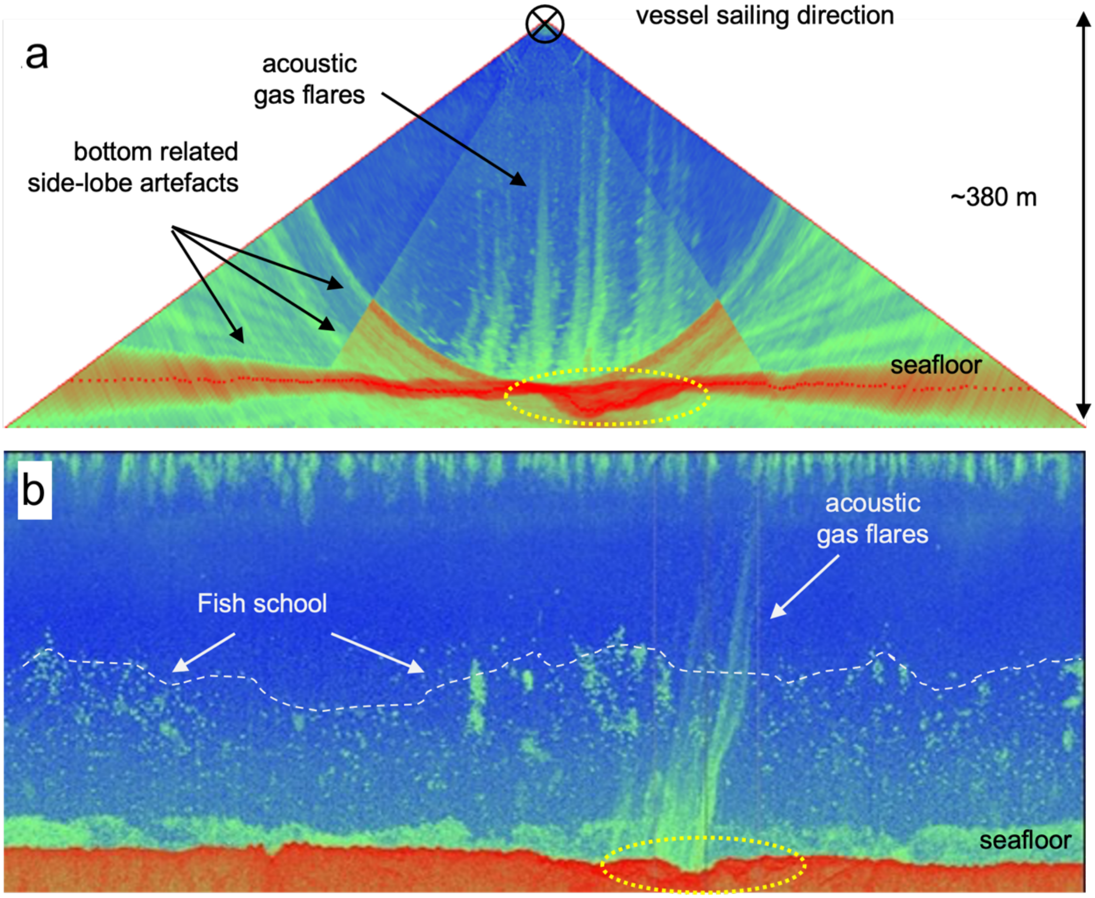

Supplementary Figure 3. The methane concentration (nmol L^{-1}) measured with the SAGE sensor from each dive. The bubble plumes observed with the ROV camera are marked with white circles. Methane concentration was measured during ROV Dive 13 (**a**), Dive 14 (**c**), Dive 15 (**b**), and Dive 16 (**d**).

The bathymetry used to plot the data was obtained from the RV Kronprins Hakon ship during the AKMA 3 expedition. The ship uses an EM 302 multibeam echo sounder with an operating frequency of 30 kHz and is designed to perform seabed mapping with high resolution and accuracy to a maximum depth of more than 7000 m. Beam focusing is applied both during reception and transmission. EM 302 is equipped with a function to reduce the transmission power to avoid disturbing mammals if they are close by. The EM 302 (including the SBP 300) is mounted in the ice window in the bottom hull of the vessel. The multibeam bathymetry data was processed and cleaned during the cruise using QPS Qimera Software. Initial grid surfaces with a 2- to 7.5 m resolution were produced for the Borealis site.

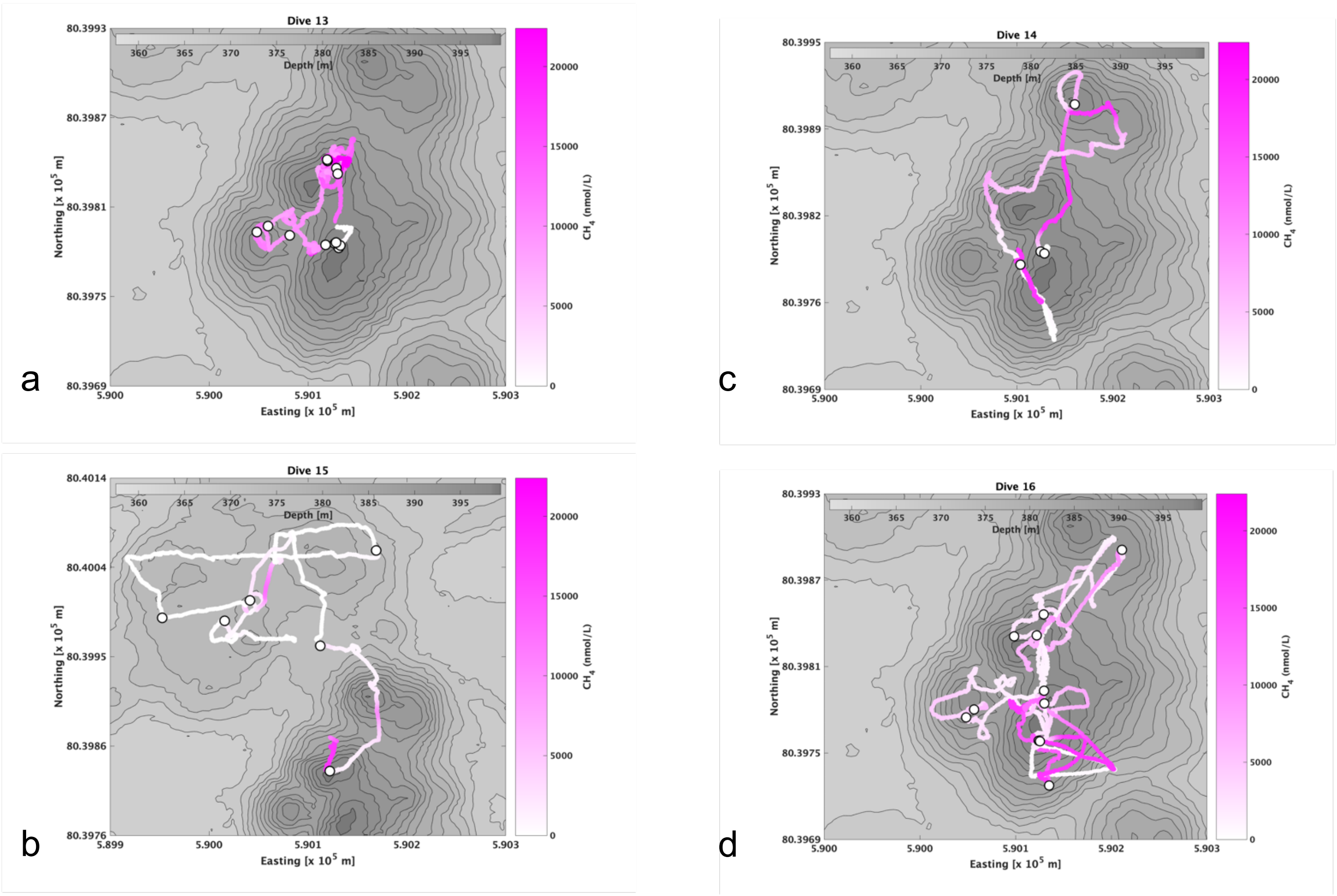

Supplementary Figure 4. Organic geochemistry data for sediment samples collected from the Borealis area. a Extract gas chromatogram showing n-alkanes distribution in a sediment sample from a pushcore. The chromatogram includes an evident hump caused by an unresolved complex mixture (UCM) which is typical of crude oil. **b** Depositional environments (indicated with boxes with different colors/patterns) inferred for the sources of oils based on dibenzothiophene (DBT)/phenanthrene (P) and Pristane (Pr)/Phytane (Ph) ratios[74]. DBP/P < 1 coupled with Pr/Ph between 1 and 3 suggest a marine shale/lacustrine source rock. **c** Age indications based on sterane C-number distribution and nordiacholestane ratio (NDR) [75]. **d** Maturity indications from hopanes isomerism suggest an early oil window maturity. **e** Maturity indications based on sterane isomerism are consistent with the ones derived from hopanes. std=standard.

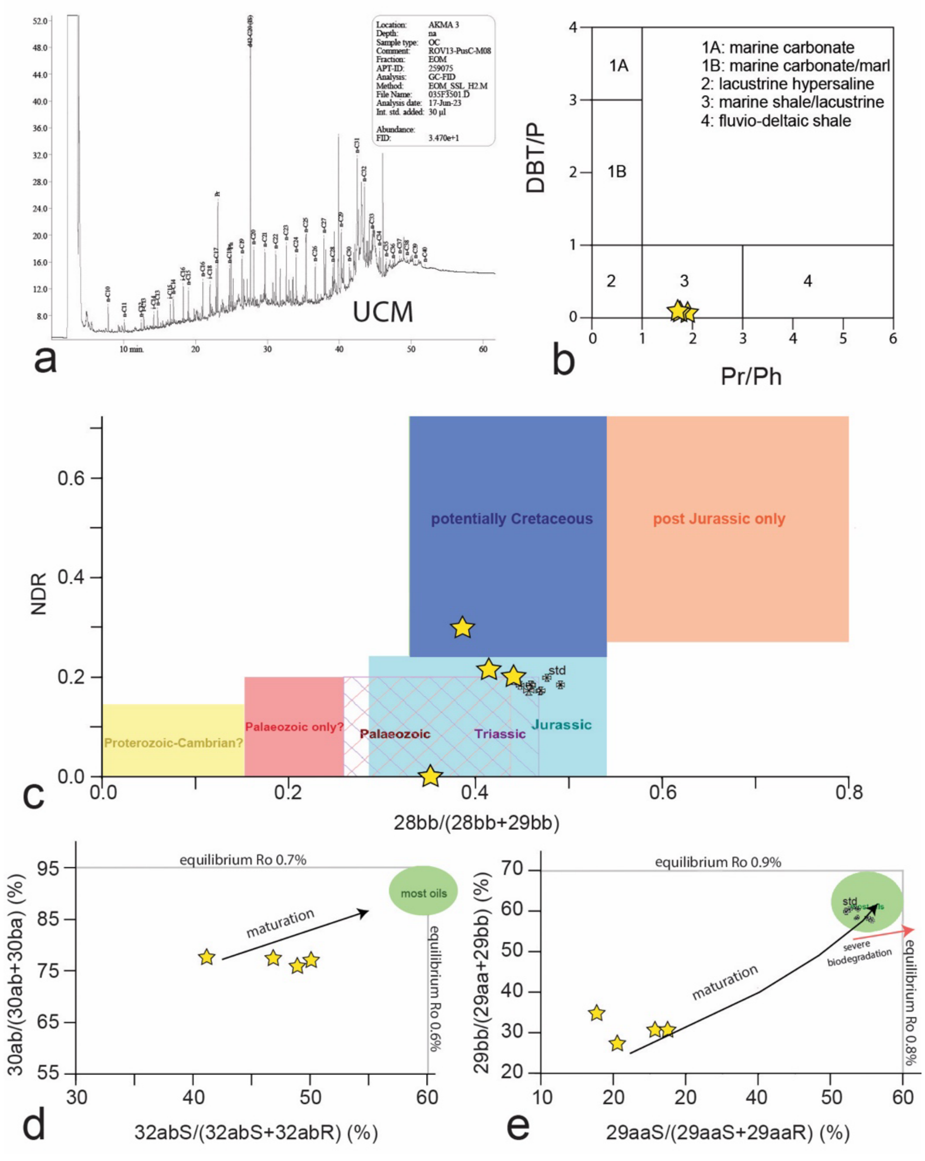

Supplementary Figure 5. ROV frame showing the sampling events. ROV frame showing the sampling events (**a** AKMA3 ROV13-BlaC01; **b** AKMA3 ROV13 - BlaC 03; **c** AKMA3 ROV16 PusC C5-M05, **d** AKMA3 ROV16-WatS-01, **e** AKMA3 ROV15 CarC 1, **f** AKMA3 ROV15 CarC 2, **g** AKMA3-ROV16-Blac1, **h** AKMA3-ROV16-Blac2, **i** AKMA3-ROV16-Blac3, **l** AKMA3-ROV16-CarC1, **m** AKMA3-ROV16-CarC2, **n** AKMA3-ROV16-Scoop) of the samples used for the different analyses presented in this paper as reported in Supplementary Data 1. The coordinates on the ROV frame indicate where the sample has been taken and are reported in decimal degrees.

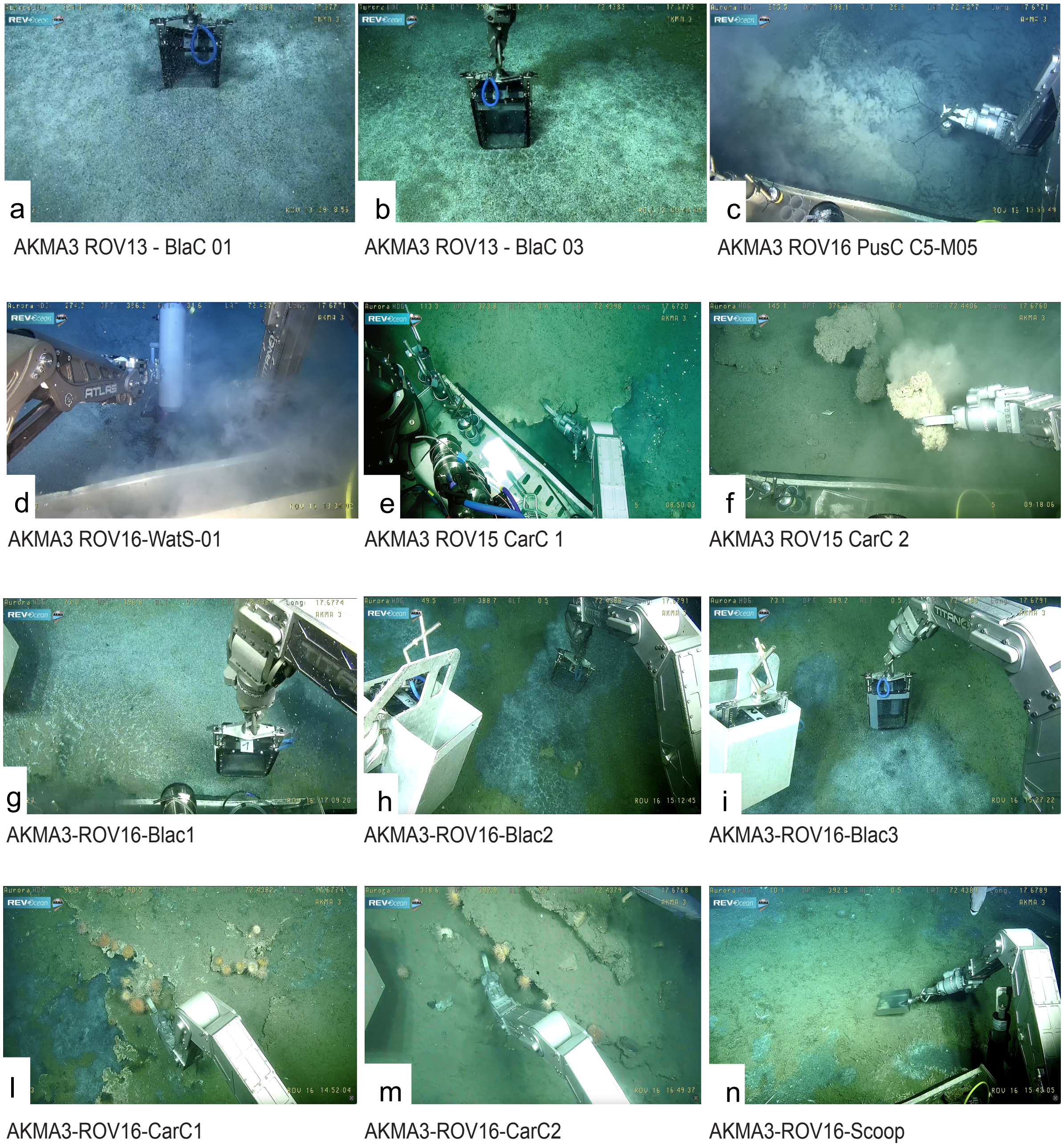

Supplementary Figure 6. High-resolution bathymetric map (with a 4-meter grid) of Borealis mud volcano. a The map details the volcano's four distinct craters located on the seafloor in correspondence with the conduit identified in the seismic random line in Supplementary Figure 1 that serves as conduits for fluid expulsion. Adjacent to the craters, a notable ploughmark is visible, indicating a past event of glacial or sedimentary activity that has left a linear depression on the seafloor. **b** Bathymetry scale (in m, vertical bar) and distance scale (in m, horizontal bar).

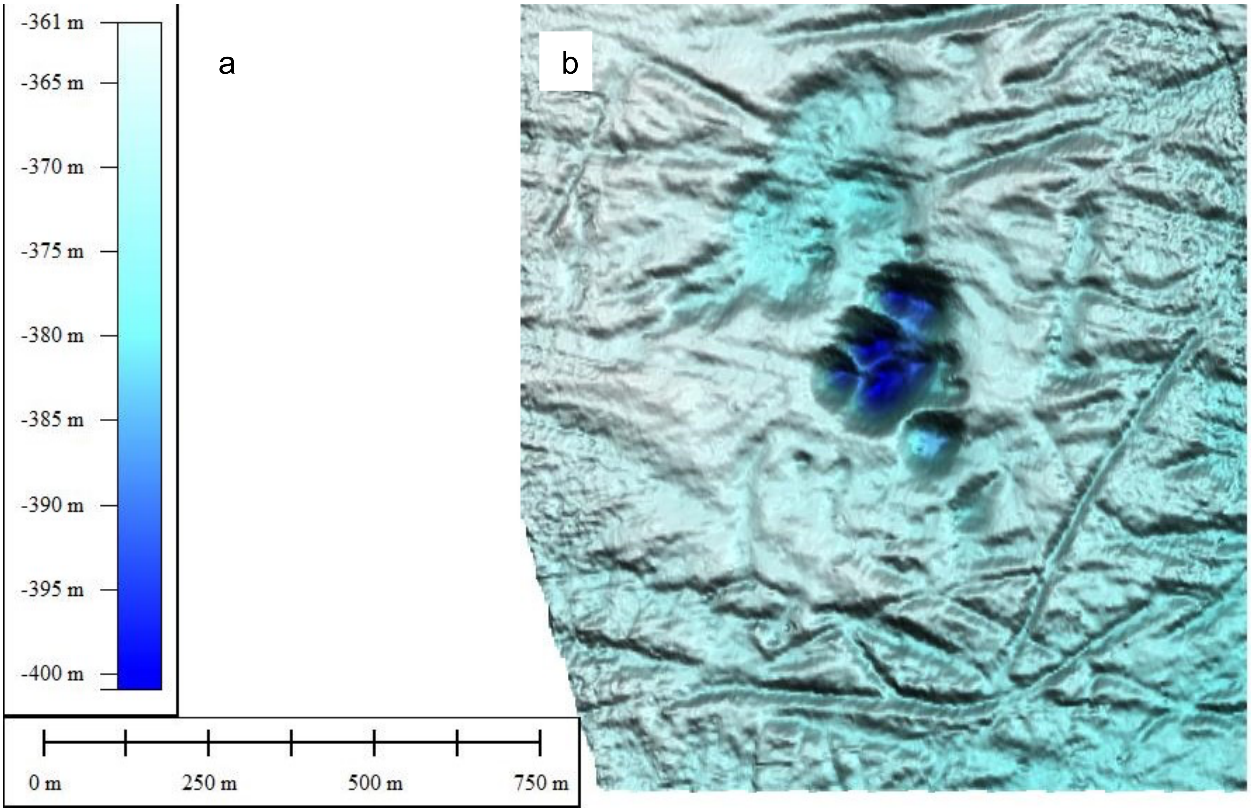

Supplementary Figure 7. ROV track lines. Map showing the track lines of the ROV Dives (13, 14, 15 and 16) in the Borealis area. The bathymetry used to plot the data was obtained from the RV Kronprins Hakon ship during the AKMA 3 expedition. The ship uses an EM 302 multibeam echo sounder with an operating frequency of 30 kHz and is designed to perform seabed mapping with high resolution and accuracy to a maximum depth of more than 7000 m. Beam focusing is applied both during reception and transmission. EM 302 is equipped with a function to reduce the transmission power to avoid disturbing mammals if they are close by. The EM 302 (including the SBP 300) is mounted in the ice window in the bottom hull of the vessel. The multibeam bathymetry data was processed and cleaned during the cruise using QPS Qimera Software. Initial grid surfaces with a 2- to 7.5 m resolution were produced for the Borealis site.

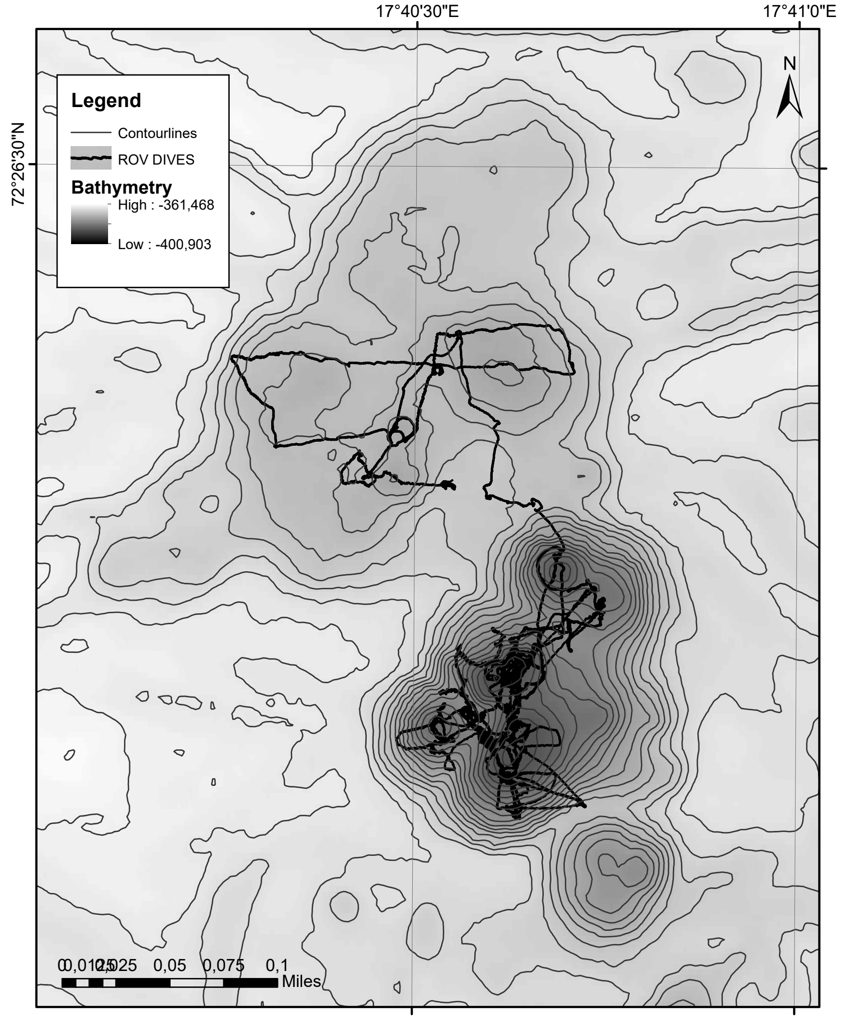

Supplement: Supplementary file 1 — Supplementary Information [file 41467_2024_55712_MOESM1_ESM.pdf]
