# Supplementary material for: Sanctuary for vulnerable Arctic species at the Borealis Mud Volcano
Source: Nat Commun. 2025 Jan 27;16:504. doi: 10.1038/s41467-024-55712-x (PMC11772567; doi:10.1038/s41467-024-55712-x)
Supplement: Supplementary file 2 — Description of Additional Supplementary Files [file 41467_2024_55712_MOESM2_ESM.pdf]

Description of Additional Supplementary Files

File Name: Supplementary Data 1
Description: **Analysed samples and types of analyses performed at the Borealis mud volcano.**

File Name: Supplementary Data 2
Description: **The methane concentration (nmol L-1) measured in water samples.** The samples were collected from the CTD Niskin bottles during AKMA3-07-CTD 84 and AKMA3-07-CTD 86 at different water depth (m below sea surface). The name of the water samples injected in the Gas Chromatographer - FID is also indicated (see Chapter 4.2, Methane measurements). The data are plotted and shown in Fig.1.

File Name: Supplementary Data 3
Description: **The methane concentration (nmol L-1) was measured in water with the SAGE sensor during AKMA3-07-Dive-13.** The table indicates the latitude and longitude, and water depth for each measurement performed during the ROV 13 (See chapter 4.2 Methane measurements, for details on the sensor). The data are plotted and shown in Figure 1 and Supplementary Figure 3. While we include here all of the raw data collected by SAGE, we note that since SAGE is capable of accurate measurements to 10,000 nmol L-1, we plot in this paper all values measured above this as ≥10,000 nmol L-1.

File Name: Supplementary Data 4
Description: **The methane concentration (nmol L-1) was measured in water with the SAGE sensor during AKMA3-07-Dive-14.** The table indicates the latitude and longitude, and water depth for each measurement performed during the ROV 14 (See chapter 4.2 Methane measurements, for details on the sensor). The data are plotted and showed in in Figure 1 and Supplementary Figure 3. While we include here all of the raw data collected by SAGE, we note that since SAGE is capable of accurate measurements to 10,000 nmol L-1, we plot in this paper all values measured above this as ≥10,000 nmol L-1.

File Name: Supplementary Data 5
Description: **The methane concentration (nmol L-1) was measured in water with the SAGE sensor during AKMA3-07-Dive-15.** The table indicates the latitude and longitude, and water depth for each measurement performed during the ROV 15 (See chapter 4.2 Methane measurements, for details on the sensor). The data are plotted and shown in Figure 1 and Supplementary Figure 3. While we include here all of the raw data collected by SAGE, we note that since SAGE is capable of accurate measurements to 10,000 nmol L-1, we plot in this paper all values measured above this as ≥10,000 nmol L-1.

File Name: Supplementary Data 6
Description: **The methane concentration (nmol L-1) was measured in water with the SAGE sensor during AKMA3-07-Dive-16.** The table indicates the latitude and longitude, and water depth for each measurement performed during the ROV 16 (See chapter 4.2 Methane measurements, for details on the sensor). The data are plotted and shown in Figure 1 and Supplementary Figure 3. While we include here all of the raw data collected by SAGE, we

note that since SAGE is capable of accurate measurements to 10,000 nmol L-1, we plot in this paper all values measured above this as ≥10,000 nmol L-1.

File Name: Supplementary Data 7
Description: **Amplicon Sequencing Variants (ASV) of foraminiferal eDNA.** For each ASV the foraminifera taxon identified on a number of reads > 100 are indicated. Raw data is available on NCBI Sequence Read Archive SRA under the accession number PRJNA1192685.

File Name: Supplementary Data 8
Description: **The abundance of the vulnerable megafauna taxa observed at Borealis MV**. Matrix showing the visualized area (expressed in m^2) of the substrates detected in the study area through video analysis and the number of main vulnerable megafauna occurrences (Redfish; Corals) identified through video analysis, per type of substrate (BG: background; MM: microbial mats; CC: carbonate crusts) and each video (ROV 13: ROV 14; ROV 15; ROV 16).
